# A new species of *Tegenaria* Latreille, 1804 (Araneae, Agelenidae) from Turkey

**DOI:** 10.3897/zookeys.51.467

**Published:** 2010-07-23

**Authors:** Rahşen S. Kaya, Kadir B. Kunt, Yuri M. Marusik, İsmail H. Uğurtaş

**Affiliations:** 1 Department of Biology, Faculty of Arts and Sciences, Uludağ University, TR-16059, Nilüfer, Bursa, Turkey; 2 Turkish Arachnological Society, Eserköy Sitesi, 9/A Blok, No 7, TR-06530, Ümitköy, Ankara, Turkey; 3 Institute for Biological Problems of the North RAS, Portovaya Str. 18, Magadan, Russia

**Keywords:** Agelenidae, new species, Tegenaria, Turkey

## Abstract

A new species of the spider genus Tegenaria Latreille, 1804 is described, based on newly collected specimens from Turkey. Detailed morphological descriptions, diagnosis and figures of the copulatory organs of both sexes are presented. Finally, a checklist and distribution maps for Turkish Tegenaria species are provided.

## Introduction

The spider family Agelenidae currently constitutes 514 species in 42 genera and has a global distribution (Platnick 2010). Tegenaria Latreille, 1804 is the largest genus of the family, with 101 described species, primarily from the Palaearctic region, but with some from the Oriental Region and a few from the Nearctic (Roth 1968; Gertsch 1971; Levy 1996; Platnick 2010). To date, 22 Tegenaria species have been reported from Turkey, of which 15 are endemic to the country (Bayram et al. 2010; Platnick 2010). Brignoli (1972, 1978a, b) was the most prominent contributor to the knowledge of Turkish agelenid spiders, having described and/or recorded 16 species from the country. However, most of these species are still poorly understood and remain known only from their original descriptions.

In general, the supraspecific taxonomy of Tegenaria and the tribe Tegenariini is poorly resolved. The genus includes species with very different palpal and epigynal conformations. Recently, Guseinov et al. (2005) removed 26 species from Tegenaria and transferred them to Malthonica Simon, 1898. The taxonomy of Tegenaria with respect to the Mediterranean fauna is currently being developed by A. Bolzern (Bolzern 2007; Bolzern et al. 2008, 2009).

During our surveys of the Turkish spider fauna, we found an undescribed species in the southern region of the country. This species possesses copulatory organs different from other Turkish and eastern Mediterranean Tegenaria and is described here as a new species.

## Material and methods

The specimens were studied using a Leica M205 C stereomicroscope. The description of colour was based on live specimens. The epigyne was macerated in 10% KOH. Measurements were taken with a micrometer eyepiece from the dorsal aspect of the palps and legs. The morphological terminology follows Levy (1996) and Guseinov et al. (2005). Leg spination follows Bolzern et al. (2008, 2009). The taxonomy and world distribution data were derived from Platnick (2010).

Specimens were photographed using an Olympus Camedia E-520 camera attached to an Olympus SZX16 stereomicroscope. Images were produced using “CombineZP” image stacking software. Photographs were taken in dishes of different sizes with paraffin at the bottom. Holes of different sizes were made in the paraffin in order to keep specimens in the required position.

The following abbreviations are used:

AERanterior eye row; ALEanterior lateral eyes; AMEanterior median eyes; PERposterior eye row; PLEposterior lateral eyes; PMEposterior median eyes.

All measurements are in millimeters (mm).

Type specimens have been preserved in 70% ethanol and deposited in the Zoological Museum of Uludağ University (ZMUU, Department of Biology, Bursa, Turkey) and the Zoological Museum of the Moscow State University (ZMMU, Moscow, Russia; curator Dr K.G. Mikhailov).

## Description

Genus Tegenaria Latreille, 1804

### Tegenaria bayrami sp. n.

urn:lsid:zoobank.org:act:705E551F-CD34-4BAE-9757-48B8AC25B970

[Fig F1] [Fig F2] Figs 1-16 20 

#### Material.

Holotype: male (ZMUU):Turkey: Antalya Province, Manavgat District, Beşkonak Village, Köprülü Canyon, 37°11’N 31°11’E, 243 m a.s.l., 01.VI.2006, R. S. Kaya.

**Figures 1-5 F1:**
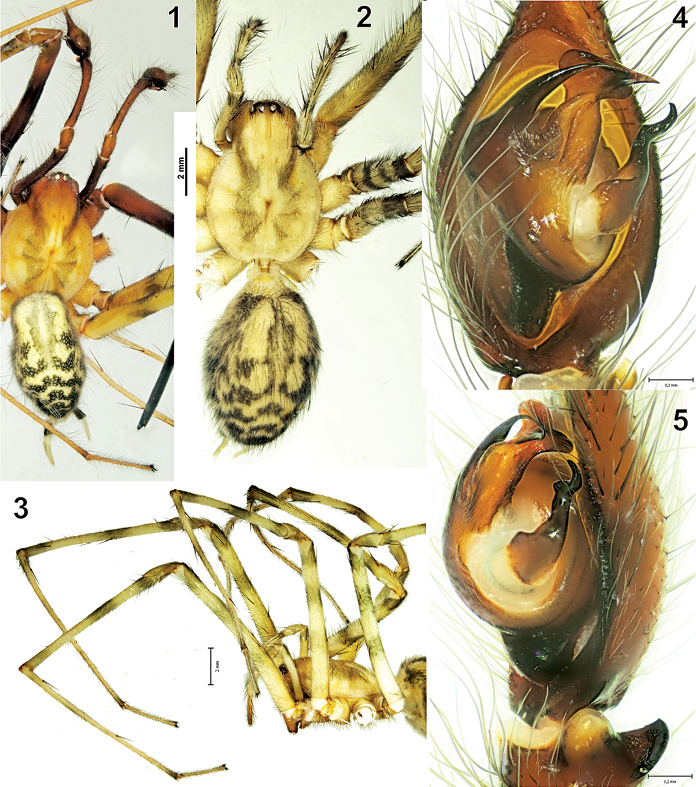
The general appearance and male palp of Tegenaria bayrami sp. n. **1** male, dorsal view **2** female, dorsal view **3** female prosoma, lateral view, showing long legs **4-5** male palp, ventral and retrolateral views respectively.

**Figures 6-10 F2:**
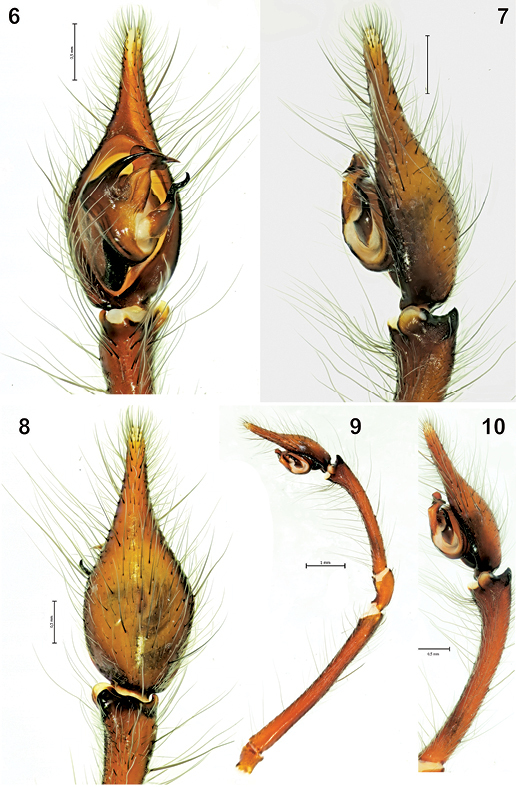
The male palp of Tegenaria bayrami sp. n. **6** ventral view **7** lateral view **8** dorsal view **9** entire palp, retrolateral view **10** tibia and tarsus, retrolateral view.

**Figures 11-16:**
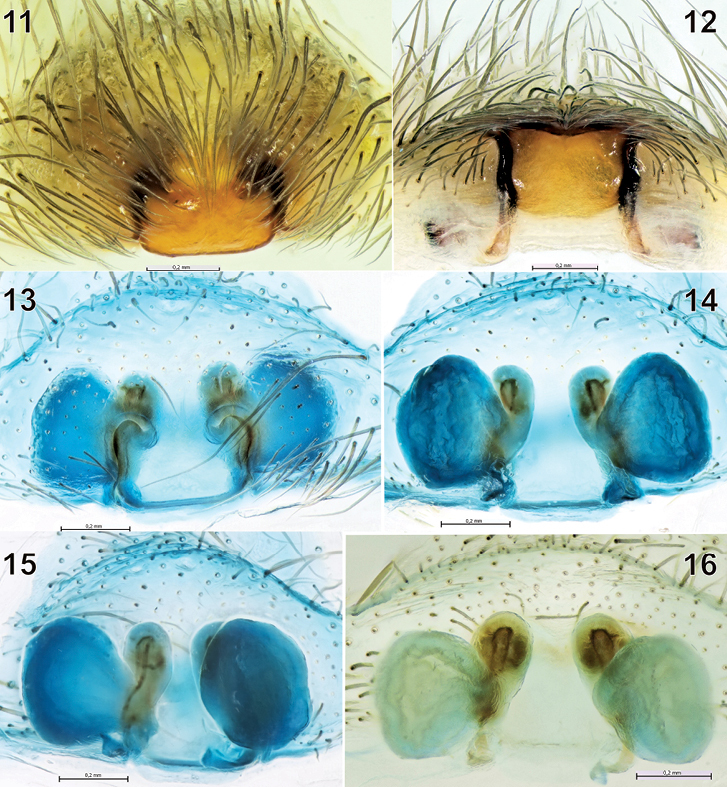
The epigyne and spermathecae of Tegenaria bayrami sp. n. **11** before maceration, ventral view **12** ditto, caudal view **13-14** after maceration, ventral and dorsal views **15** ditto, latero-dorsal view **16** ditto, dorsal view.

#### Paratypes.

One male, one female and three subadult females (ZMUU), one male (ZMMU): same data as for the holotype, 21.VI.2010.

#### Etymology.

The species is named in honor of Prof. Dr. Abdullah Bayram, who has made an important contribution to Turkish arachnology.

#### Diagnosis.

Tegenaria bayrami sp. n. is closely related to Tegenaria longimana Simon, 1898, Tegenaria vignai Brignoli, 1978 and Tegenaria halidi Guseinov, Marusik & Koponen, 2005. Males of the new species can be easily distinguished from the mentioned species by the shape of the large and curved median apophysis and the pointed tip of the conductor (Figs 4, 6). The female of Tegenaria bayrami sp. n. can be separated from all other Tegenaria species by the square-shaped epigynal plate (Figs 11), short ducts and round spermathecae (Figs 14–16).

#### Description.

##### Male (holotype).

Total length 9.00. Prosoma: carapace 4.60 long, 3.50 wide. Carapace brownish yellow, with two longitudinal darkened bands (Fig 1), margins not darkened, scarce plumose hairs present. Cephalic region: 1.80 wide, darker and separated from the thoracic region by a distinct, darkened line. PER: 0.9 wide. Diameter of PME: 0.20; PLE: 0.22; AME: 0.10; ALE: 0.22. Distance of PME–PME: 0.10; PME–AME: 0.07. Eye formula: ALE=PLE>PME>AME. Clypeus height (measured from bottom of AME): 0.35, clypeus height (measured from bottom of ALE): 0.30. Clypeus deep reddish brown. Eye rows: AER slightly recurved, PER straight in dorsal view. Eye region darker. Chelicerae: 1.17 long; 0.90 wide. Chelicerae red-brown. Gnathocoxae: 1.32 long; 0.65 wide. Labium: 0.77 long; 0.70 wide. Gnathocoxae and labium brown. Sternum: 2.25 long; 2.22 wide. Sternum heart-shaped, pointed backwards, brown, with a light median band and three pairs of sublateral round spots. Legs light brown, with dark annulations, densely covered with plumose hairs; legs I and II are darker than legs III and IV (Fig. 1). Number of dorsal tarsal trichobothria on tarsi I and III: 10, tarsi II and IV: 9. Leg measurements are given in Table 1, and spine formulae in Table 2. Abdomen: 4.4 long, 2.6 wide; dorsum appears yellowish brown, with a reticulate patter of a series of transverse black lines along the dorsal mid-line and sides. Venter pale brown, with many short hairs and longitudinal black bars between the epigastric furrow and spinnerets.

Male palp as in Figs 4–10; very long (femur 5.10, patella 1.00, tibia 2.70, tarsus 2.20, (total 11.00), longer than body. Femur approximately 1.8 times longer than tibia, tibia 1.2 times longer than cymbium. Retrolateral tibial apophysis with two branches: lateral branch in dorsal view elongated and more or less rectangular, in retrolateral view triangular and tapering off towards the tip; broad and rounded latero-ventral branch with a small protuberance close to lateral branch in retrolateral view. Median apophysis long, in ventral view its base is large and broad, tip is claw-like. Conductor long, beak-shaped in ventral view; embolus thick and short in ventral view.

##### Female.

Total length 11.40. Prosoma: carapace 5.40 long, 4.00 wide. Carapace light yellow, with two longitudinal darkened bands (Figs 2–3), margins not darkened, scarce hairs present. Cephalic region: 2.40 wide, darker and separated from the thoracic region by a distinct, darkened line. PER: 1.17 wide. Diameter of PME: 0.20; PLE: 0.22; AME: 0.12; ALE: 0.22. Distance of PME–PME: 0.20; PME–AME: 0.25. Eye formula: ALE=PLE>PME>AME. Clypeus height (measured from bottom of AME): 0.42, clypeus height (measured from bottom of ALE): 0.40. Clypeus dark brown. Eye rows: AER slightly recurved, PER straight in dorsal view. Chelicerae: 2.50 long; 1.30 wide. Chelicerae brown. Gnathocoxae: 1.50 long; 0.90 wide. Labium: 0.90 long; 0.80 wide. Gnathocoxae and labium brown, labium slightly longer than wide. Sternum: 2.60 long; 2.40 wide. Sternum heart-shaped, pointed towards rear end, brown, with light median band and three pairs of sublateral round spots. Legs light brown, with dark annulations, densely covered by long hairs, plumose hairs present. Number of dorsal tarsal trichobothria on tarsi I and IV: 11, tarsi II and III: 10. Leg measurements are given in Table 1, and spine formulae in Table 2. Abdomen: 6.00 long, 4.10 wide; dorsum yellowish brown, with a reticulate patter of a series of transverse, thick black lines along the dorsal mid-line and sides (Fig. 2). Venter pale brown, with many short hairs and longitudinal black bars between the epigastric furrow and spinnerets.

**Table 1:** Leg and palp measurements of the holotype male and paratype female of Tegenaria bayrami sp. n.

		femur	patella	tibia	metatarsus	tarsus	total
male	palp	5.1	1.0	2.7	-	2.2	11.0
	Leg I	11.1	2.1	11.6	13.6	4.2	42.6
	Leg II	9.7	1.9	9.6	12.0	3.6	36.8
	Leg III	8.2	1.8	7.8	10.5	3.0	31.3
	Leg IV	9.6	1.8	9.4	13.4	3.4	37.6
female	palp	3.3	1.2	2.2	-	2.7	9.4
	Leg I	9.7	2.1	8.8	11.4	3.7	35.7
	Leg II	8.8	2.0	7.9	9.6	3.2	31.5
	Leg III	7.2	1.6	6.6	8.5	3.0	26.9
	Leg IV	9.4	2.0	8.2	11.1	3.2	33.9

**Table 2:** Spination of legs and palps of Tegenaria bayrami sp. n. The formula gives the number of spines in the following order: dorsal – prolateral – retrolateral – ventral. The letter ‘p’ indicates a pair of spines that occur at this position.

		femur	patella	tibia	metatarsus	tarsus
palp	Paratype (female)	2-1-1-0	2-0-0-0	2-2-0-0	-	many
leg I	Holotype (male)	1-2-3-0	1-0-0-0	0-1-1-1	0-1-0-1p+1+1p	0-0-0-0
	Paratype (female)	2-3-2-0	2-0-0-0	1-1-1-1	1-1-1-1p+1p+1p	0-0-0-0
leg II	Holotype (male)	2-3-2-0	1-0-0-0	1-2-1-1+1p	0-2-1-1p+1+1p	0-0-0-0
	Paratype (female)	2-3-2-0	1-0-0-0	1-2-0-1p+1p	0-2-1-1p+1p+1p	0-0-0-0
leg III	Holotype (male)	2-2-2-0	1-0-0-0	1-2-1-1p+1p+1p	1-3-3-1p+1+1+1p	0-0-0-0
	Paratype (female)	4-5-4-0	1-0-0-0	1-2-1-1p+1p+1p	1-4-4-1p+1+1+1p+1p	0-0-0-0
leg IV	Holotype (male)	2-2-3-0	1-0-0-0	1-2-2+1p+1p	2-3-4-1+1+1+1p	0-0-0-0
	Paratype (female)	2-2-3-0	2-0-0-0	2-3-2-1p+1+1+1p	2-3-4-1p+1p+1p	0-0-0-0

Epigyne and spermathecae as in Figs 11-16. Fovea absent, median plate square-shaped; copulatory openings almost invisible on intact epigyne, but readily visible following hair removal. Insemination duct short, spermathecae almost round.

#### Habitat.

The new species was collected from damp places of rocky areas along the River Köprüçay (Köprülü Canyon, Antalya). The canyon is located on the lower slopes of the West Taurus Mountain ranges. Samples were collected from their big funnel webs during the day. The collection of a male in copula with a female clearly suggests that both sexes described here are conspecific.

#### Distribution.

Turkey, known only from the type locality (Fig. 20).

## Checklist of Tegenaria species known from Turkey

Figs 17-20

### 1. Tegenaria agnolettii

Brignoli, 1978

Fig. 20

Tegenaria agnolettii Brignoli 1978a: 44, fig. 7 (known from female only).

#### General distribution:

Turkey.

#### Distribution in Turkey:

Antalya Province: Döşemealtı District, Mustanini Cave (Brignoli 1978a).

### 2. Tegenaria agrestis

(Walckenaer, 1802)

Fig. 17 

#### General distribution:

Europe to Central Asia, USA and Canada.

#### Distribution in Turkey:

Anatolia, no exact locality. It was reported from Turkey by Caporiacco (1935) only. It is likely that this record is the result of a misidentification.

For a complete list of references see Platnick (2010).

### 3. Tegenaria atrica

C.L. Koch, 1843

Fig. 18 

#### General distribution:

Europe, introduced to North America.

#### Distribution in Turkey:

İstanbul Province: Şile District; Kayseri Province: Yeşilhisar District, Harmankaya Cave (Roewer 1959). It is likely that this species was misidentified from Turkish specimens and probably does not occur in the country.

For a complete list of references see Platnick (2010).

### 4. Tegenaria averni

Brignoli, 1978

Fig. 20

Tegenaria averni Brignoli 1978a: 50, fig. 10 (known from female only).

#### General distribution:

Turkey.

#### Distribution in Turkey:

Mersin Province: Silifke District, Cennet Cave (Brignoli 1978a).

### 5. Tegenaria bayrami sp. n.

Fig. 20 

#### General distribution:

Turkey only.

#### Distribution in Turkey:

Antalya Province: Manavgat District, Beşkonak Village, Köprülü Canyon.

### 6. Tegenaria bithyniae

Brignoli, 1978

Fig. 19

Tegenaria bithyniae Brignoli 1978b: 515, fig. 97 (known from female only).

#### General distribution:

Bulgaria and Turkey.

#### Distribution in Turkey:

Bolu Province: Abant (Brignoli 1978b).

### 7. Tegenaria comnena

Brignoli, 1978

Fig. 20

Tegenaria comnena Brignoli 1978b: 520, fig. 108 (known from female only).

#### General distribution:

Turkey.

#### Distribution in Turkey:

Trabzon Province: Maçka District, Sümela Monastery (Brignoli 1978b).

### 8. Tegenaria cottarellii

Brignoli, 1978

Fig. 20

Tegenaria cottarellii Brignoli 1978b: 523, fig. 106 (known from female only).

#### General distribution:

Turkey.

#### Distribution in Turkey:

Rize Province: Kalkandere District; Artvin Province: Borçka District (Brignoli 1978b).

### 9. Tegenaria domestica

(Clerck, 1757)

Fig. 17 

#### General distribution:

Cosmopolitan, synanthropic in most places.

#### Distribution in Turkey:

Hatay Province: Narlıca Town, Narlıca Cave; Urfa Province; Mardin Province: Midyat District, a hill near Derömer Area (Roewer 1959); Kırıkkale Province (Bayram et al. 2005).

For a complete list of references see Platnick (2010).

### 10. Tegenaria elysii

Brignoli, 1978

Fig. 20

Tegenaria elysii Brignoli 1978a: 49, fig. 9 (known from female only).

#### General distribution:

Turkey.

#### Distribution in Turkey:

Mersin Province: Silifke District, Dilek Cave and Cennet Cave (Brignoli 1978a).

### 11. Tegenaria faniapollinis

Brignoli, 1978

Fig. 20

Tegenaria faniapollinis Brignoli 1978a: 50, fig. 13 (known from female only).

#### General distribution:

Turkey.

#### Distribution in Turkey:

Hatay Province: Harbiye Town, Harbiye Cave (Brignoli 1978a).

### 12. Tegenaria forestieroi

Brignoli, 1978

Fig. 20

Tegenaria forestieroi Brignoli 1978a: 45, fig.12 (known from female only)

#### General distribution:

Turkey.

#### Distribution in Turkey:

Konya Province: Beyşehir District, Kurucuova Village (Asarini Cave and İnönüini Cave); Seydişehir District (Ferzene Cave and fossile sinkhole of Tınaztepe Cave); Çamlık District (Körükini Cave); Hadım District (Suçıktığı Cave); Antalya Province: Akseki District (Demirci Dükkanları Cave, a cave in Dikmen Village and from a well) (Brignoli 1978a).

### 13. Tegenaria hamid

Brignoli, 1978

Fig. 20

Tegenaria hamid Brignoli 1978b: 515, fig. 96 (known from female only).

#### General distribution:

Turkey.

#### Distribution in Turkey:

Isparta Province: Eğirdir District (Brignoli 1978b).

### 14. Tegenaria karaman

Brignoli, 1978

Fig. 20

Tegenaria karaman Brignoli 1978a: 48, fig. 8, (known from female only).

#### General distribution:

Turkey.

#### Distribution in Turkey:

Konya Province: Seydişehir District, Ferzene Cave (Brignoli 1978a).

### 15. Tegenaria longimana

Simon, 1898

Fig. 19 

#### General distribution:

Turkey, Georgia and Russia.

#### Distribution in Turkey:

Rize Province: Fındıklı District (50 km NE of Rize, towards Hopa); Artvin Province: Hopa District; Trabzon Province: Maçka District, Altındere Village, Sümela Monastery (Brignoli, 1978b).

For a complete list of references see Platnick (2010).

### 16. Tegenaria mamikonian

Brignoli, 1978

Fig. 20

Tegenaria mamikonian Brignoli 1978b: 520, fig. 104 (known from female only).

#### General distribution:

Turkey.

#### Distribution in Turkey:

Artvin Province: Yalnızçam Pass (Brignoli 1978b).

### 17. Tegenaria melbae

Brignoli, 1972

Fig. 20

Tegenaria melbae Brignoli 1972: 171, Figs 19–20 (known from female only).

#### General distribution:

Turkey.

#### Distribution in Turkey:

Diyarbakır Province: Lice Province, Korkha Cave (Brignoli 1972).

### 18. Tegenaria parietina

(Fourcroy, 1785)

Fig. 17 

#### General distribution:

Europe, North Africa to Central Asia, Uruguay and Argentina.

#### Distribution in Turkey:

İstanbul Province (Pavesi 1876; Karol 1966); Ankara Province (Karol 1966); Mersin Province: Tarsus District, Gülek Town (Topçu et al. 2006), Bursa Province: Görükle Campus (Kaya and Uğurtaş 2007)

For a complete list of references see Platnick (2010).

### 19. Tegenaria percuriosa

Brignoli, 1972

Fig. 20 

#### General distribution:

Turkey.

#### Distribution in Turkey:

Isparta Province: Aksu District, Anamas Plateau, Zindan Cave (Brignoli 1972, 1978a; Gasparo 2007) and Barla (Brignoli, 1978a); Konya Province: Beyşehir, Hacı Akif Cave (Brignoli, 1978a); Bolu Province: Abant (Brignoli 1978b); Sivas Province: Çamlıbel Pass (Brignoli, 1978b); Ankara Province: Kızılcahamam District (Brignoli, 1978b) and Antalya Province: Alanya District, Dim Cave (Kunt et al. 2008)

For a complete list of references see Platnick (2010).

### 20. Tegenaria rhodiensis

Caporiacco, 1948

Fig. 19

Tegenaria rhodiensis Brignoli 1978b: 513, Figs 90–93.

#### General distribution:

Rhodes and Turkey.

#### Distribution in Turkey:

Konya Province: Beyşehir District, Beyşehir Lake, Island of Hacı Akif; Isparta Province: Eğirdir District (Brignoli 1978b).

### 21. Tegenaria tekke

Brignoli, 1978

Fig. 20

Tegenaria tekke Brignoli 1978b: 516, fig. 98 (known from female only).

#### General distribution:

Turkey.

#### Distribution in Turkey:

Antalya Province: Around Kaş District (Brignoli 1978b).

### 22. Tegenaria vignai

Brignoli, 1978

Fig. 20

Tegenaria vignai Brignoli 1978b: 524, Figs 110–112, 114.

#### General distribution:

Turkey.

#### Distribution in Turkey:

Artvin Province: Borçka District (Brignoli 1978b).

### 23. Tegenaria xenophontis

Brignoli, 1978

Fig. 20

Tegenaria xenophontis Brignoli 1978b: 522, Figs 103, 105 (known from female only).

#### General distribution:

Turkey.

#### Distribution in Turkey:

Trabzon Province: Maçka District (Sümela Monastery) and Zigana Pass (Brignoli 1978b).

**Figure 17:**
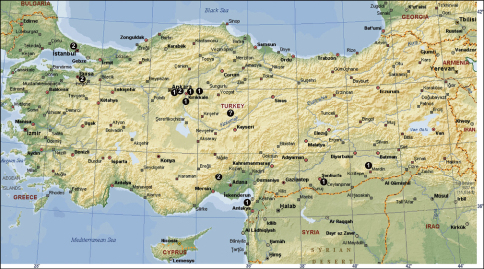
The distribution of Tegenaria agrestis (? = no exact locality in Anatolia; see Caporiacco, 1935), Tegenaria domestica (**1**) and Tegenaria parietina (**2**) in Turkey.

**Figure 18:**
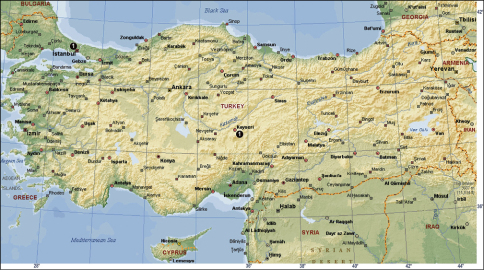
The distribution of Tegenaria atrica (**1**) in Turkey.

**Figure 19:**
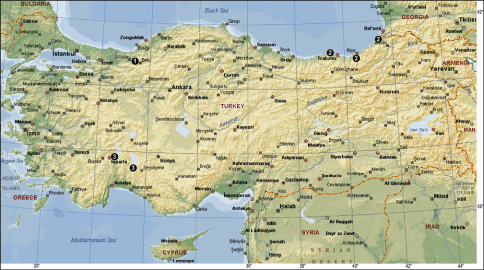
The distribution of Tegenaria bithyniae (**1**), Tegenaria longimana (**2**) and Tegenaria rhodiensis (**3**) in Turkey.

**Figure 20:**
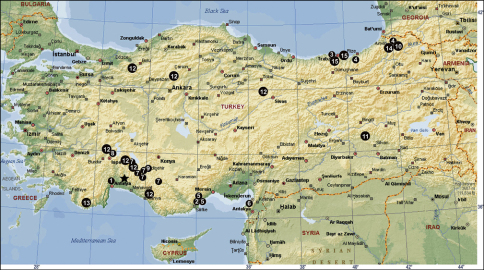
The distribution of Tegenaria agnolettii (**1**), Tegenaria averni (**2**), Tegenaria comnena (**3**), Tegenaria cottarellii (**4**), Tegenaria elysii (**5**), Tegenaria faniapollinis (**6**), Tegenaria forestieroi (**7**), Tegenaria hamid (**8**), Tegenaria karaman (**9**), Tegenaria mamikonian (**10**), Tegenaria melbae (**11**), Tegenaria percuriosa (**12**), Tegenaria tekke (**13**), Tegenaria vignai (**14**), Tegenaria xenophontis (**15**) and Tegenaria bayrami sp. n. (star) in Turkey.

## Discussion

Twenty-three Tegenaria species have now been reported from Turkey, including the newly described species. Only four of them (Tegenaria agrestis, Tegenaria atrica, Tegenaria domestica and Tegenaria parietina) have broad distribution ranges, whereas 16 species are endemic to Turkey. Among the species restricted to Turkey or to the eastern Mediterranean, 14 are known exclusively from females and only four species are known from both sexes. All species known from only one sex were described by Brignoli (1972; 1978 a,b). Reasoning from the illustrations of Brignoli (1972; 1978 a,b) and knowing that the epigyne of Tegenaria is rather simple, it can be assumed that some of Brignoli’s species names will be synonymized in the future. Nevertheless, the diversity of Tegenaria in Turkey is very high compared to other regions. The Turkish fauna includes more species than some well studied and species-rich countries as Italy and Spain (15 species of Tegenaria in each) (Helsdingen 2009). Neighbouring Bulgaria has only 12 species reported (Deltshev 1995). Of course, it is possible that some of the species known from females may belong to the closely related genus Malthonica (sensu Guseinov et al. 2005). If one compares the species diversity of the genera Tegenaria and Malthonica, the species richness of both in Turkey (31 species) is higher than in other countries: 27 in continental Italy, 23 in Bulgaria and France, and 22 in Spain.

Although the currently known diversity of Tegenaria and Malthonica in Turkey is already extraordinarily high, the actual diversity may be even higher. Many caves, a favourite habitat for Tegenaria, have never been studied or sampled for spiders in Turkey. Therefore, we expect that more new species will be found in the future.

## Supplementary Material

XML Treatment for Tegenaria bayrami

XML Treatment for Tegenaria agnolettii

XML Treatment for Tegenaria agrestis

XML Treatment for Tegenaria atrica

XML Treatment for Tegenaria averni

XML Treatment for Tegenaria bayrami

XML Treatment for Tegenaria bithyniae

XML Treatment for Tegenaria comnena

XML Treatment for Tegenaria cottarellii

XML Treatment for Tegenaria domestica

XML Treatment for Tegenaria elysii

XML Treatment for Tegenaria faniapollinis

XML Treatment for Tegenaria forestieroi

XML Treatment for Tegenaria hamid

XML Treatment for Tegenaria karaman

XML Treatment for Tegenaria longimana

XML Treatment for Tegenaria mamikonian

XML Treatment for Tegenaria melbae

XML Treatment for Tegenaria parietina

XML Treatment for Tegenaria percuriosa

XML Treatment for Tegenaria rhodiensis

XML Treatment for Tegenaria tekke

XML Treatment for Tegenaria vignai

XML Treatment for Tegenaria xenophontis

